# A Case of Dissociative Identity Disorder in CAMHS: At the Intersection of Neurodivergence, Trauma, and Culture

**DOI:** 10.1192/bjo.2026.11770

**Published:** 2026-06-30

**Authors:** Jiaying Chen

**Affiliations:** Oxford Health NHS Foundation Trust, Oxford, United Kingdom

## Abstract

**Aims:**

Dissociative identity disorder (DID) is characterised by the existence of two or more distinct identities within an individual, affecting their consciousness, behaviour, and memory. Accounting for only 1% of the psychiatric population, it is a rare and often controversial diagnosis. This case report describes the diagnosis of DID in a neurodivergent adolescent from an ethnic minority background, whose assessment was complicated by layers of complexities. Through his journey within the mental health services in England, the successes and challenges in supporting the needs of young people in similar circumstances will be discussed. All identifying details have been anonymised.

**Methods:**

Joseph is a 14-year-old asylum seeker of Black ethnicity who arrived in the UK at age 9. Known to the mental health service from age 13 for low mood, he was reviewed following a suicide attempt by jumping from a bridge. Joseph, however, stated that it was not him but ‘Karl’ who took the leap. This behaviour was initially hypothesised to be part of autistic thinking or passivity phenomena of an emerging psychotic episode. Joseph continued to describe being in distinct personality states across various contexts, and his mother corroborated episodes of dissociation involving behavioural changes and memory disruption. A diagnosis of DID was made following multiple specialist assessments.

**Results:**

Joseph’s care involved multi-agency collaboration between a range of specialist teams across the public sector. The Crisis team provided a swift response and intensive follow-up during periods of escalated risks. The Outreach team, which specialises in engaging young people who are difficult to reach, built rapport with Joseph at home. Educational and social care agencies played active roles in navigating uncertainties regarding Joseph’s legal status. Engaging with a young autistic person required persistence and skill. Understanding the family’s cultural and religious backgrounds was crucial for differentiating culturally congruent experiences from those that were pathological and distressing. A key barrier to planning management from the formulation was the gaps in services and training to address such intricate presentation holistically.

**Conclusion:**

While DID may not be a commonly encountered diagnosis, the context which this case brings is a familiar one: ethnic minorities constitute 18% of the UK population; autism is diagnosed in 3% of adolescents; dissociative disorders have a prevalence of 10% in the clinical population and are strongly associated with trauma. Resource allocation for training professionals to become trauma-informed, culture-aware, and neurodivergence-sensitive across mental health and non-mental health services would therefore be essential.

